# The Lipid Profiles in Different Characteristics of Women with PCOS and the Interaction Between Dyslipidemia and Metabolic Disorder States: A Retrospective Study in Chinese Population

**DOI:** 10.3389/fendo.2022.892125

**Published:** 2022-07-04

**Authors:** Fei Guo, Zhentao Gong, Taniya Fernando, Lingshan Zhang, Xiaoyong Zhu, Yingli Shi

**Affiliations:** ^1^ Obstetrics and Gynecology Hospital of Fudan University, Shanghai, China; ^2^ Shanghai Medical College of Fudan University, Shanghai, China; ^3^ Shanghai Key Laboratory of Female Reproductive Endocrine Related Diseases, Shanghai, China

**Keywords:** dyslipidemia, lipid profiles, hyperandrogenism, insulin resistance, dysglycemia, PCOS

## Abstract

**Purpose:**

To exhibit the lipid profiles in PCOS women with different characteristics and to access correlations between alternation of key lipid parameters and characteristics of PCOS.

**Design:**

A retrospective study.

**Participants:**

A total of 700 PCOS women were included.

**Methods:**

Retrospective study on 700 women (age 24.6 ± 4.7 years), diagnosed with PCOS in the outpatient department of Obstetrics and Gynecology Hospital of Fudan University according to Rotterdam criteria. Anthropometric features, hormone levels, lipid levels, and metabolic parameters were measured and compared between PCOS patients with different characteristics.

**Results:**

There was a high prevalence of dyslipidemia among Chinese PCOS patients (41.3%), and the most common pattern was low HDL. Patients with clinical hyperandrogenism presented with significantly decreased HDL and Apo-A levels. The levels of TG, LDL, Apo-B, TG/HDL, and Apo-B/Apo-A were significantly increased in the insulin resistance subgroup. The levels of TC and TG were significantly increased in the dysglycemia and T2DM women. And in general, the levels of TG, and Apo-B had an increasing trend with BMI. Moreover, AI, TG/HDL, and Apo-B/Apo-A ratios were associated with some characteristics of PCOS, such as insulin resistance, and obesity.

**Conclusion:**

The PCOS women with different characteristics presented with different lipid profiles, and there is a complex correlation between lipid metabolism and PCOS characteristics, which may explain the increased risk of long-term cardiovascular disease. Regular screening of blood lipids is essential for PCOS women. Identification of optimal subgroups in PCOS patients that need lipid-lowering treatment and therapeutic effectiveness is worth exploring.

## Introduction

Polycystic ovary syndrome (PCOS) is a common endocrine disease (1, 2), affecting between 6 to 22% of women worldwide. Its cardinal features include ovulatory dysfunction, hyperandrogenism (HA), and polycystic changes in the ovary (3). It is often diagnosed in the reproductive phase of life when women with PCOS are confronted with infertility, irregular menstruation, or symptoms of hyperandrogenism, including acne, androgenic alopecia, and hirsutism. In addition, dyslipidemia is increasingly common in young adult women with PCOS (4). Retrospective and prospective cohort studies further indicate an increase in coronary atherosclerotic cardiopathy risk in PCOS (5–8). However, the potential interactions of endocrine disorders and metabolic abnormalities on lipid profiles in PCOS patients have not been studied extensively. Hyperandrogenism, insulin resistance (IR), impaired glucose metabolism, and obesity may exert independent, as well as interrelated effects on circulating lipid profiles, the underlying mechanism also requires further explanation.

In this article, we addressed the differences in blood lipid and lipoprotein profiles between different characteristics of PCOS and intended to discuss the association between changes in lipid parameters and abnormal metabolism states. Identification of the subpopulation with a higher risk of developing lipid metabolic and cardiovascular disease is important since these groups may require closer metabolic monitoring.

## Materials and Methods

### Subjects

Our study enrolled 700 PCOS patients, aged 12 to 38, diagnosed in the outpatient department of Obstetrics and Gynecology Hospital of Fudan University from 2018 to 2021. All these subjects met the revised 2003 Rotterdam diagnostic criteria for PCOS (9). The diagnosis of adolescent PCOS (under 18 years old) was relatively strict. The presence of all Rotterdam diagnostic criteria, including clinical and/or biochemical evidence of hyperandrogenism (after the exclusion of other pathologies), persistent oligomenorrhea for at least two years, and polycystic appearance of the ovary are mandatory in the diagnosis of adolescent PCOS. The exclusion criteria included other causes of hyperandrogenism (cushing syndrome, congenital adrenal hyperplasia, or androgen-secreting tumors), hyperprolactinemia, hypogonadotropic hypogonadism, thyroid dysfunction, premature ovarian failure, a previously diagnosed type 2 diabetes mellitus (T2DM), pregnancy, lactation period, treatment with insulin-sensitizing drugs and hormone drugs (contraceptives, antiandrogens, or glucocorticoids) 6 months before the beginning of the research. Patients had no clinically evident acute or chronic diseases such as infection, or tumors. No smokers or alcoholics. They were recommended to stay a healthy diet and lifestyle. All study participants gave their informed consent.

### Assessment of Reproductive Endocrine, Blood Lipids, and Metabolic Parameters

Bodyweight, barefoot height, and waist circumference (WC) were measured at the outpatient department and body mass index (BMI, kg/m^2^) was calculated. Hirsutism was assessed with the Ferriman-Gallwey score (F-G score) (10). Blood samples were obtained at random from amenorrhoeic women or on the 3-5th days of the menstrual cycle from menstruating women. The serum lipid levels were measured in the morning after overnight fasting. The oral glucose tolerance test (OGTT) and insulin release test (IRT) were performed after a 12-hour fast, with glucose and insulin measurements in the basal sample, and at 0.5, 1, 2, and 3-hour intervals after the glucose load. The samples were stored at 4°C and immediately transferred to the clinical laboratory.

Hormone parameters: follicular stimulating hormone (FSH), luteinizing hormone (LH), testosterone (T), sex hormone-binding globulin (SHBG), and anti-müllerian hormone (AMH), levels were assessed using an Access immune luminescence analyzer (Beckman DXI800, USA). The concentration of total cholesterol (TC), triglycerides (TG), high-density lipoprotein cholesterol (HDL), low-density lipoprotein cholesterol (LDL), apolipoprotein A (Apo-A), apolipoprotein B (Apo-B), and free fatty acid (FFA) was measured by enzymatic colorimetric method with the 7600 autoanalyzer (Hitachi 7600, Japan). Blood glucose was measured by the glucose oxidase method (Hitachi 7600, Japan). Serum insulin was determined by a microparticle enzyme immunoassay using an AxSYM system (Abbott Laboratories, Dainabot Co., Japan). All analyses were performed by the experienced physician of the hospital laboratory. Insulin resistance was calculated using the homeostatic model assessment (HOMA-IR) (11). HOMA-IR = fasting insulin (uU/ml) × fasting glucose (mmol/L)/22.5. Free androgen index (FAI%) = T(ng/ml) ×100/SHBG (nmol/L). Arteriosclerosis index (AI) =(TC-HDL)/HDL.

### Definition of Dyslipidemia, Hyperandrogenism, Insulin Resistance (IR), Dysglycemia, and Diabetes

In our study, dyslipidemia patients met the standard for Chinese adult dyslipidemia diagnosis (TC≥6.2 or TG≥2.3 or LDL≥4.1 or HDL<1.0 mmol/l). All the PCOS women were allocated into four subgroups. Clinical hyperandrogenism (clinical-HA) included patients with clinical manifestations, such as acne, androgenic alopecia, and hirsutism but with normal T levels. The biochemical hyperandrogenism (biochemical-HA) was defined as T≥0.7ng/ml and without hyperandrogenic clinical manifestations. People who have neither clinical-HA nor biochemical-HA were classified as non-HA group. IR group included the women with acanthosis nigricans or HOMA-IR>2.6, and the rest of the participants were listed in the non-IR group. According to the results of OGTT, fasting blood glucose level exceeding 6.1mmol/L was defined as impaired fasting glucose, and the 2-hour blood glucose levels exceeding 7.8mmol/L were defined as impaired glucose tolerance. They were both categorized as dysglycemia. Conversely, the rest of the people were listed in the normal glucose group. Patients with a fasting blood glucose level of 7.0mmol/L or higher, a 2-hour blood glucose level of 11.1mmol/L or higher, or a random blood glucose level exceeding 11.1mmol/L accompanied by typical diabetes symptoms were categorized as the diabetes group, and the rest of the patients were listed in the non-diabetes group. Moreover, according to BMI and age, they were distributed to different subgroups (BMI<18.5, 18.5-24.9, 25-29.9, and ≥30), (age ≤18, 19-25, 26-30, and ≥35 years).

### Statistical Analysis

The data were analyzed using the Statistical Package for Social Sciences (SPSS, version 21). We assessed the normality of the distribution of all continuous variables using the F-test. The Student’s T-test was used to compare the mean values of both groups. One-way analysis of variance (ANOVA) was used to compare the mean levels of three or more groups. A *Post Hoc* test [least significance difference (LSD) and Tamhane’s T2 (M)] performed following ANOVA was used to determine the statistical significance of the difference between these groups. Logistic regression analysis was used to correct the confounding variables. X^2^ test was used to compare prevalence rates between different groups. Pearson correlation analysis was performed to define the correlations between lipid levels and various characteristics of PCOS. For all analyses, a P-value of < 0.05 was considered statistically significant.

## Results

### Types of Dyslipidemia in PCOS Patients

Among all the recruited women in our research, 289 met the standard of dyslipidemia, accounting for 41.3%.**Table 1** showed various patterns of dyslipidemia described in PCOS, including hypercholesterolemia, hypertriglyceridemia, low HDL, and combined hyperlipidemia, which were highly atherogenic and predispose to cardiovascular disease (5, 6). Low HDL was the most common type, accounting for 57.4% of all dyslipidemia patients.

**Table 1:** Dyslipidemia patterns in PCOS women.

Dyslipidemia Patterns	n (%)
Hypercholesterolemia	14 (4.8)
Hypertriglyceridemia	71 (24.6)
Low high-density lipoproteinemia	166 (57.4)
Combined Hyperlipidemia	38 (13.2)

### Clinical Characteristics, Hormonal Levels, and Metabolic Profiles in the Normal Lipid and Dyslipidemia Groups

As shown in **Table 2**, there were no differences between the normal lipid and dyslipidemia groups regarding age, BMI, and WC. In terms of the hormone levels, FSH and T levels were both lower in the dyslipidemia group after adjusting for age, BMI, and WC. However, the Ferriman-Gallwey scores and FAI levels were not different in the two groups. There were no differences between the two groups concerning the levels of serum glucose recorded in the OGTT. Meanwhile, the fasting and the third hour of insulin levels during the IRT significantly increased in the dyslipidemia group (13.67 vs. 12.05, P=0.004) and (44.33 vs.36.48, P=0.033), as well as HOMA-IR (3.05 vs. 2.65, P=0.009). After adjusting for age, BMI and WC, the differences continued to exist.

**Table 2:** Clinical and biochemical characteristics of PCOS women according to blood lipid states.

	Dyslipidemia group (n = 289)	Normal Lipid group (n = 411)	P	P^a^
Age	24.22 ± 5.03	24.90 ± 4.45	0.064	
BMI	24.95 ± 5.05	24.60 ± 4.81	0.373	
WC	87.63 ± 13.15	86.55 ± 12.62	0.389	0.479
F-G Score	1.28 ± 1.34	1.03 ± 1.54	0.024	0.751
FSH (mIU/ml)	6.69 ± 1.51	7.09 ± 1.68	0.005	0.003*
LH (mIU/ml)	12.57 ± 6.94	13.27 ± 7.28	0.226	0.320
T (ng/ml)	0.76 ± 0.27	0.82 ± 0.26	0.002	0.004*
SHBG (nmol/L)	37.00 ± 29.79	42.11 ± 30.44	0.092	0.259
AMH (ng/ml)	9.38 ± 5.30	9.31 ± 4.53	0.888	0.448
FAI	3.41 ± 4.43	3.04 ± 2.43	0.297	0.584
LH/FSH	1.93 ± 1.08	1.91 ± 1.07	0.779	0.933
OGTT (mmol/L) 0h	5.03 ± 1.36	4.89 ± 0.75	0.109	0.208
0.5h	8.48 ± 4.62	8.23 ± 1.45	0.391	0.696
1h	8.55 ± 3.64	8.44 ± 2.36	0.632	0.959
2h	6.88 ± 2.61	6.73 ± 2.10	0.396	0.386
3h	6.68 ± 11.84	5.34 ± 6.53	0.089	0.075
INR (uU/ml) 0h	13.67 ± 8.64	12.05 ± 7.82	0.013	0.004*
0.5h	97.70 ± 63.67	92.12 ± 55.80	0.230	0.634
1h	115.69 ± 72.99	108.42 ± 67.13	0.195	0.773
2h	101.00 ± 74.48	90.79 ± 66.57	0.071	0.399
3h	44.33 ± 49.71	36.48 ± 40.43	0.032	0.033*
HOMA-IR	2.90 ± 1.94	2.56 ± 1.63	0.021	0.009*

Values are shown as mean ± standard deviation. BMI, Body mass index; WC, waist circumference; F-G score, Ferriman–Gallwey score; FSH, Follicle stimulating hormone; LH, Luteinizing hormone; T, Testosterone; SHBG, Sex hormone-binding globulin; AMH, Anti-Mullerian hormone; FAI, Free androgen index; OGTT, Oral glucose tolerance test; IRT, Insulin release test; HOMA-IR, Homeostatic model assessment for insulin resistance. P ^a^: adjusted age, BMI, and WC.

P*: The differences were considered significant.

### Comparison of the Blood Lipid Profiles in PCOS Patients With Different Clinical Characteristics

**Figure 1:**
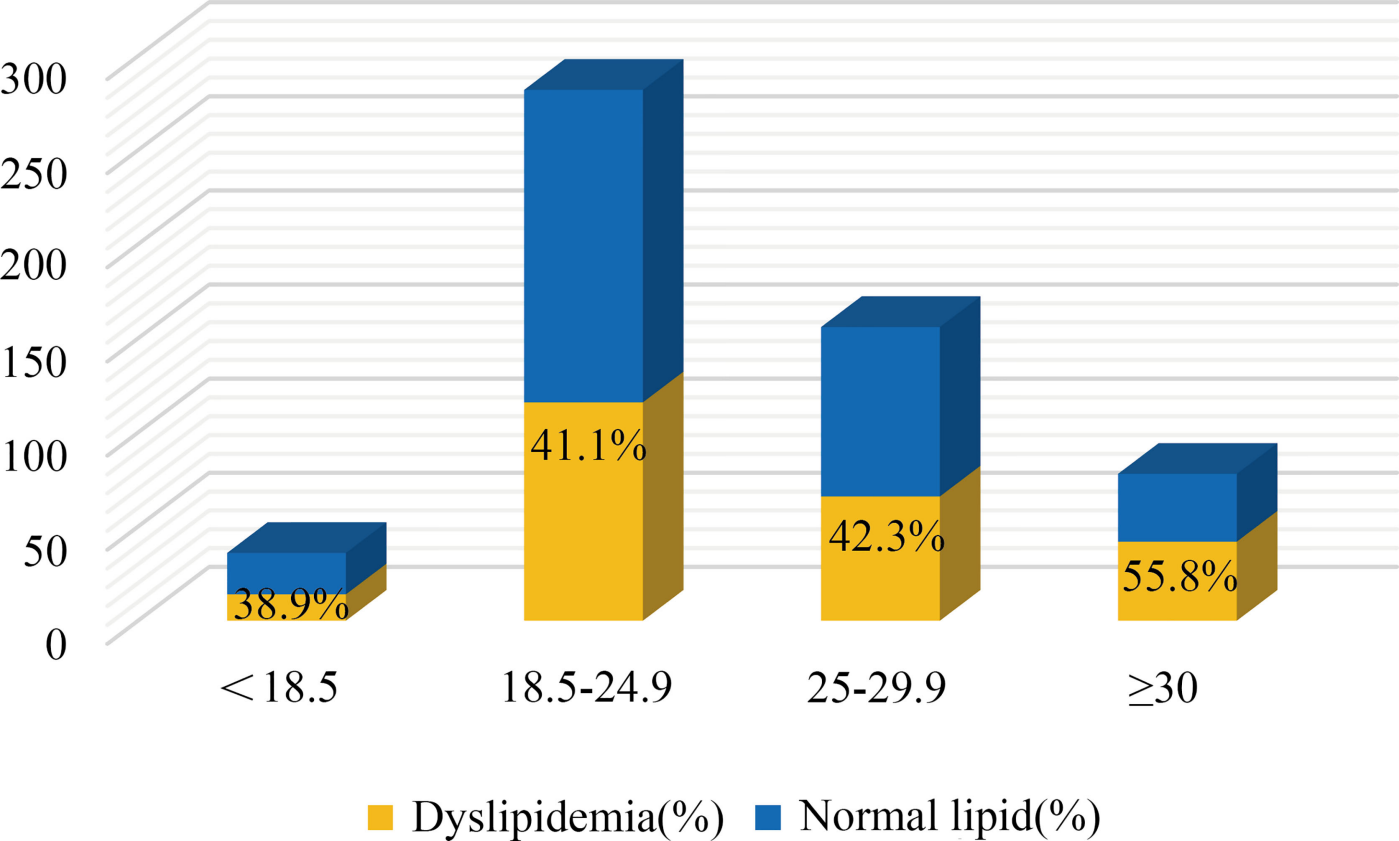
The distribution of patients with dyslipidemia in different BMI subgroups. P = 0.220.

Hyperandrogenism (clinical and biochemical), IR, dysglycemia, and obesity are all notable features of PCOS women. Among these PCOS patients, 23.4% were clinical hyperandrogenism, 35.8% were biochemical hyperandrogenism, 54.0% were insulin resistance, 22.9% were dysglycemia, 6.3% were diabetes, and 42.4% were overweight. There was no difference in lipid profiles between biochemical-HA group and non-HA group after adjusting for age, BMI, and WC (data not shown), but the levels of HDL and Apo-A significantly decreased in the patients with clinical-HA, as noted in **Table 3A**. The mean levels of TG, LDL, and Apo-B were significantly increased in women with IR, as well as the ratios TG/HDL and Apo-B/Apo-A. We discovered that TC levels significantly increased in the dysglycemia group after correction. Meanwhile, the level of TG significantly increased in the diabetes group compared to their non-diabetes counterpart. We further calculated the odds ratios (ORs) and found that insulin resistance and clinical hyperandrogenism were risk factors for dyslipidemia in PCOS patients, OR=2.202 (95%CI:1.585-3.059, P=0.000) and OR=1.744 (95%CI: 1.008-3.015, P=0.046) noted in **Table 3B**. The mean levels of TG, Apo-B, TG/HDL, and Apo-B/Apo-A exhibited an increasing tendency with BMI in the former three subgroups. But in the BMI≥30 subgroup, there was no different from those with BMI=25-29.9 patients, **Table 3C**. In terms of the prevalence of dyslipidemia, there were no differences in the four BMI subgroups (P=0.220), **Figure 1**. We found that the levels of TG, HDL, Apo-B, AI, TG/HDL, and Apo-B/Apo-A also presented an increasing trend with age, but they were not statistically significant as shown in **Table 3C**.

**Table 3A:** Comparison of serum lipid profiles in PCOS patients with or without hyperandrogenism, insulin resistance, dysglycemia, and diabetes.

	Clinical-HA	non-HA	P	P^a^	IR	non-IR	P	P^a^	Dysglycemia	Normal Glucose	P	P^a^	Diabetes	non-Diabetes	P	P^a^
Age	23.44	25.80	0.000		24.94	23.81	0.002		25.39	24.14	0.005		25.33	24.37	0.213	
BMI	24.63	24.72	0.801		27.26	21.30	0.000		26.53	23.94	0.000		28.66	24.39	0.000	
WC	86.87	87.45	0.898		93.40	77.47	0.000		92.72	84.65	0.000		99.56	86.30	0.000	
TC (mmol/L)	4.45	4.65	0.093	0.298	4.82	4.61	0.004	0.124	4.98	4.63	0.000	0.015*	5.02	4.70	0.033	0.698
TG (mmol/L)	1.57	1.48	0.632	0.479	1.86	1.01	0.000	0.003*	1.76	1.36	0.001	0.372	2.22	1.43	0.000	0.024*
LDL (mmol/L)	2.60	2.28	0.005	0.830	2.36	2.33	0.701	0.025*	2.42	2.32	0.315	0.059	2.42	2.35	0.646	0.112
HDL (mmol/L)	1.21	2.02	0.000	0.008*	1.88	1.76	0.213	0.468	1.97	1.76	0.095	0.414	2.07	1.79	0.243	0.364
Apo-A (g/L)	1.09	1.28	0.000	0.025*	1.15	1.28	0.000	0.438	1.16	1.22	0.044	0.748	1.14	1.21	0.208	0.872
Apo-B (g/L)	0.91	0.95	0.271	0.162	1.01	0.84	0.000	0.001*	1.02	0.90	0.000	0.492	1.13	0.92	0.000	0.143
FFA (mmol/L)	0.56	0.68	0.074	0.093	1.85	0.99	0.156	0.133	2.34	1.19	0.250	0.337	2.37	1.37	0.422	0.619
AI	3.30	2.99	0.004	0.065	3.26	2.94	0.000	0.318	3.31	3.07	0.000	0.428	3.25	3.12	0.273	0.202
TG/HDL	1.48	1.27	0.216	0.223	1.66	0.88	0.000	0.006*	1.56	1.23	0.003	0.487	1.85	1.28	0.002	0.070
Apo-B/Apo-A	0.89	0.80	0.045	0.785	0.94	0.71	0.000	0.007*	0.95	0.80	0.000	0.132	1.07	0.83	0.000	0.097

BMI, Body mass index; WC, waist circumference; TC, Total cholesterol; TG, Triglycerides; LDL, Low-density lipoprotein; HDL, High-density lipoprotein; Apo-A, Apoprotein A; Apo-B, Apoprotein B; FFA, Free fatty acid; AI, Atherosclerosis index.

P*: The differences were considered significant.

**Table 3B:** Association between insulin resistance, clinical hyperandrogenism, dysglycemia, and dyslipidemia in PCOS women.

	Odds Ratio	95%CI	P
Insulin resistance	2.202	1.585 - 3.059	0.000*
Clinical-HA	1.744	1.008 – 3.015	0.046*
Dysglycemia	1.336	0.912 - 1.958	0.137

Clinical-HA, Clinical hyperandrogenism.

P*: The differences were considered significant.

**Table 3C:** Comparison of lipid parameters in women with PCOS according to body mass index and age groups.

	BMI<18.5	18.5-24.9	25-29.9	≥30	Age ≤18	19-25	26-30	≥35
TC (mmol/L)	4.444	4.654	4.73	4.98^12^	4.43^bcd^	4.70^a^	4.80^a^	4.91^a^
TG (mmol/L)	0.79^234^	1.23^134^	1.80^12^	2.05^12^	1.24^d^	1.38	1.52	2.06^a^
LDL (mmol/L)	2.47	2.49	2.37	2.48	2.46	2.35	2.42^d^	2.03^c^
HDL (mmol/L)	1.34^3^	1.57	1.81^1^	1.83	1.51^d^	1.77^d^	1.80^d^	2.36^abc^
Apo-A (g/L)	1.22	1.22^34^	1.12^2^	1.08^2^	1.17	1.21	1.21	1.25
Apo-B (g/L)	0.79^234^	0.88^134^	1.01^12^	1.10^12^	0.84^cd^	0.91	0.97^a^	1.05^a^
FFA (mmol/L)	2.24	1.00	1.98	2.28	0.58	1.35	1.78	1.54
AI	3.15	3.09^34^	3.23^2^	3.42^2^	3.06	3.10	3.12	3.27
TG/HDL	0.75^234^	1.09^134^	1.64^12^	1.85^12^	1.18	1.24	1.32	1.80
Apo-B/Apo-A	0.68^234^	0.78^134^	0.95^12^	1.09^12^	0.77^c^	0.82	0.87^a^	0.89

TC, Total cholesterol; TG, Triglycerides; LDL, Low-density lipoprotein; HDL, High-density lipoprotein; Apo-A, Apoprotein A; Apo-B, Apoprotein B; FFA, Free fatty acid; AI, Atherosclerosis index. ^1^P < 0.05 vs group BMI<18.5; ^2^P < 0.05 vs group BMI=18.5-24.9; ^3^P < 0.05 vs group BMI=25-29.9; ^4^P < 0.05 vs group BMI≥30. ^a^P < 0.05 vs group age ≤ 18; ^b^P < 0.05 vs group age=19-25; ^c^P < 0.05 vs group age=26-30; ^d^P < 0.05 vs group age≥35.

### Correlation Analysis Between the Blood Lipid Levels and Physiological Characteristics or Biochemical Indexes of PCOS Patients

**Figure 2:**
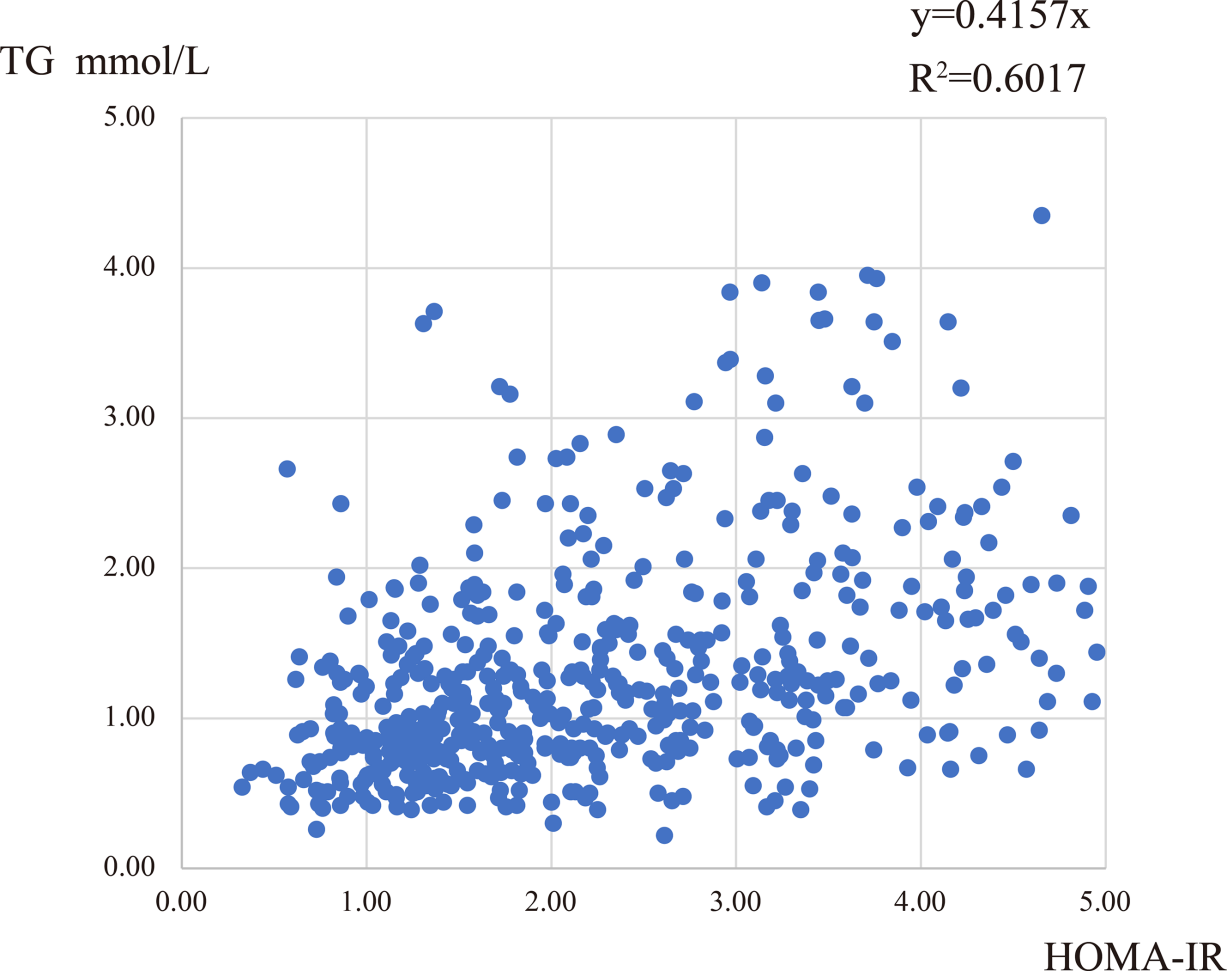
The correlation between TG and HOMA-IR.

To explore the degree of correlation between the blood lipid levels and different features in PCOS patients, we performed a correlation analysis between lipid levels and age, BMI, WC, FAI, OGTT-0h, 2h, fasting insulin, and HOMA-IR. We found that TG, Apo-B, and TG/HDL levels were positively correlated with many factors, especially HOMA-IR values, **Table 4**. Though the r values were comparatively small, these conclusions existed consistently. The correlation between TG level and HOMA-IR was approximately linear, R^2^ = 0.6017 (**Figure 2**). In addition, the levels of TC, LDL, HDL, and FFA had a weak correlation with other characteristics in PCOS women, which could be considered irrelevant (data not shown).

**Table 4:** Correlations between lipid parameters and HOMA-IR in PCOS women.

	Unadjusted		Adjusted ^a^	
	r	P	r	P
TC (mmol/L)	0.175	0.000	0.160	0.002*
TG (mmol/L)	0.433	0.000	0.378	0.000*
LDL (mmol/L)	-0.089	0.029	0.029	0.579
HDL (mmol/L)	0.185	0.000	0.121	0.022*
Apo-A (g/L)	-0.082	0.044	0.075	0.154
Apo-B (g/L)	0.309	0.000	0.204	0.000*
FFA (mmol/L)	0.018	0.653	0.014	0.785
AI	0.145	0.000	0.016	0.761
TG/HDL	0.455	0.000	0.335	0.000*
Apo-B/Apo-A	0.263	0.000	0.096	0.068

HOMA-IR, Homeostasis model assessment of insulin resistance; TC,Total cholesterol; TG, Triglycerides; LDL, Low-density lipoprotein; HDL, High-density lipoprotein; Apo-A, Apoprotein A; Apo-B, Apoprotein B; FFA, Free fatty acid; AI, Atherosclerosis index. P^a^: adjusted for age, BMI, and WC.

P*: The differences were considered significant.

## Discussion

Our study compared the blood lipid profiles with different characteristics of PCOS patients in China using a moderate sample size. The major findings of our study: (I) There was a high prevalence of dyslipidemia in Chinese PCOS patients (41.3%), and the most common was decreased HDL, accounting for 57.4%; (II) The lipid metabolic disorders were more evident in patients with clinical hyperandrogenism, and the dyslipidemia profiles were also observed in PCOS women with IR; (III) PCOS patients with impaired glucose metabolism were accompanied by dyslipidemia, indicating a close relationship between glucose and lipid metabolism; (IV) In general, the ratios of TG/HDL, and Apo-B/Apo-A were correlated with IR, with an increasing trend with BMI, which may partially explain the higher risk of cardiovascular diseases presented in PCOS patients.

In our study, 41.3% of PCOS patients suffered from dyslipidemia. But in other studies, there was a comparatively higher prevalence of dyslipidemia in the Chinese population, with 52.96% and 53.1% (12, 13), which could be attributed to the different diagnostic criteria for dyslipidemia. Compared with the 70% reported in foreign research (14), the overall prevalence of dyslipidemia was lower in the Chinese PCOS population. The most common type of dyslipidemia was low HDL, which was similar to the conclusions of other studies (13, 15). Hyperandrogenism was a common characteristic of PCOS, 82.2% were found to be clinical or biochemical hyperandrogenism. Hyperandrogenism affected women’s cardiovascular health, as it was positively correlated with blood pressure (16). At present, there were different opinions about the effect of androgen on blood lipid in PCOS. Some retrospective studies found that the lipid profiles were not associated with hyperandrogenism in PCOS women (17, 18), however, another research pointed out that hyperandrogenism was associated with a 1.5-2 fold increase in the dyslipidemia rate (19). We speculated that the poor accuracy of the current androgen detection methods may give rise to opposing conclusions. Although mass spectrometry was the most accurate detection method for testosterone, it was not used widely in clinical practice. Hence, clinical hyperandrogenism may be a better indicator of androgen activity in the body than biochemical hyperandrogenism. We divided HA into two categories: clinical hyperandrogenism, and biochemical hyperandrogenism. We found that the clinical hyperandrogenism group presented with dyslipidemia patterns, with significantly decreased HDL and Apo-A levels. Accordingly, we suggested a detailed classification of hyperandrogenemia in PCOS patients in future studies, which may enable a new discovery. In addition, extra attention should be paid to the lipid metabolic health in women with clinical hyperandrogenism.

Another interesting finding in our research was the lower FSH level in the dyslipidemia group after correction for age, BMI, and WC. The circulating dyslipidemia environment exerted a negative impact on the hypothalamic-pituitary part of the reproductive axis. Santoro reported that exposure of normal-weight women to excess lipid/insulin resulted in decreased gonadotropin secretion, including decreased LH pulse amplitude and mean FSH levels (20). Elevated lipid levels combined with hyperinsulinemia suppressed FSH secretion, supporting the association between the detrimental metabolic environment, endocrine disorders, and ovulation dysfunction in PCOS.

The dyslipidemic women often presented with hyperinsulinemia and IR (21). We found that elevated TG, LDL, and Apo-B were the main manifestations of the dyslipidemia profiles in PCOS patients with IR. Furthermore, some scholars suggested the ratios of TG/HDL and TC/HDL as strong preliminary predictors of IR in PCOS women (22, 23). There was a complex connection between hyperinsulinemia, IR, and lipid metabolism in PCOS patients. Following the ingestion of a meal containing fat, it underwent lipolysis in the intestinal lumen, then products of lipolysis entered the enterocytes *via* passive diffusion or specific transporters (24). Lipolysis, taking the elevated TG as an example, TG could be hydrolyzed in two steps or completely hydrolyzed by hormone-sensitive lipase (HSL) with the release of two FFAs and one glycerol. However, HSL was inactivated by insulin, thus inhibiting the lipolysis procedures (25–27). On the other hand, the process of lipid synthesis involved fatty acid esterification by reacting with glycerol-3-phosphate (G3P) (25). In adipose tissue, the main source of G3P was glucose generated by glycolysis (28), and this process could be promoted by insulin, and further increased lipid synthesis (25). IR was also associated with an increase in hepatic triglyceride lipase, which could accelerate the clearance of HDL, and reduce HDL levels (29). However, in our research, there was no difference in HDL levels recorded in the IR subgroups.

Some lipid parameters (TG and TC) were increased significantly in women with impaired glucose metabolism. The hyperglycemic status increased *de novo* lipogenesis (30), in turn, TG acted as the substrate to promote endogenous glucose production, hypercholesterolemia was also related to the prolonged increase in insulin levels (31). HDL could stimulate the secretion of glucagon (32). In PCOS patients, impaired glucose metabolism had an increased tendency to develop diabetes (33). In addition, many studies also proved that T2DM was often comorbid with lipid abnormalities (34, 35) and the increased atherosclerotic cardiovascular disease risk in patients with diabetes was attributed to abnormalities in lipid metabolism (36). For example, the excessive plasma LDL could be phagocytosed by macrophages and intruded into sub-endothelium, involved in the cascade to atherosclerosis (37). But in our study, the higher level of LDL was not significant in both dysglycemia and diabetes subgroups, which could be attributed to the small total number of patients (n=160 and n=44). In addition, more studies are needed to gain further insight into the precise mechanism of lipid disorders underlying hyperinsulinemia-insulin resistance-dysglycemia-T2DM in PCOS and to design better treatments for dysmetabolism.

PCOS was associated with excess adiposity, and 30%-60% of PCOS women were reported to be overweight (38). A prospective cohort study indicated that obesity contributed to the unfavorable lipid profiles in PCOS women, especially weight gain in early adulthood (39). Klop suggested that the hallmark of dyslipidemia in obesity was hypertriglyceridemia (40). In our research, the dyslipidemia profiles were also characterized by elevated TG in PCOS women with higher BMI. We further discovered that the TG levels presented an increasing tendency with BMI. Under the obese status, adipocytes became hypertrophic and released higher levels of FFA into circulation, increasing FFA uptake and lipid synthesis in the liver. Increased influx of FFA to the liver, leading to hepatic accumulation of TG (40). At the same time, increased accumulation of FFAs and their metabolite in the adipocyte led to ectopic lipid accumulation (41) and insulin resistance in the liver (42). Obesity was accompanied by macrophage infiltration into the adipose tissue, followed by a switch of their phenotype from anti-inflammatory to pro-inflammatory (43, 44). The increasing levels of inflammatory factors suppressed lipoprotein lipase (LPL) activity, contributing to hypertriglyceridemia (45). The cross-talk between adipocytes and macrophages accumulated in adipose also led to insulin resistance by blunting insulin action (46, 47). Further, obesity could exacerbate the risk of cardiovascular diseases in PCOS (48–50). Another prospective study demonstrated a 5-fold higher prevalence of subclinical coronary atherosclerosis in young obese PCOS women (51).

In our study, age didn’t significantly correlate with the dyslipidemic status in PCOS patients. Only the levels of TG, HDL, and Apo-B slightly increased in the senior groups compared with those under 18 years old. Another observational cross-sectional study pointed out that TG tended to increase over time in PCOS (52). The concentrations of TG in obese PCOS patients started to rise from the second decade of life, while the HDL levels began to decrease starting from their third decade of life (53). But the HDL level slightly increased in our study, which was probably due to the small sample size of the people older than 30 (n=74). Another cross-sectional study found no evidence for increased lipid levels in middle-aged women (mean age 50.5) with PCOS compared with the control population (54). No prospective studies have investigated the alternation of lipid profiles with age in PCOS patients. Only a few small longitudinal studies elucidated the effect of age on menstruation and hormone levels of PCOS patients (55–57). Large prospective studies were required urgently to provide a definitive answer about the alternation of lipid profiles in PCOS.

With increasing interest in lipid profiles of PCOS patients, more and more guidelines recommend screening women with PCOS for lipid patterns (58, 59). The lipid profile assessment is recommended in all PCOS patients, and the glucose tolerance and lipid status should be evaluated every 2–3 years. In addition to the blood lipid parameters, attention to the apolipoprotein in lipid profiles is also critical for PCOS patients in the long-term follow-up. Because we discover that the level of Apo-B is correlated with many features, especially fasting insulin and IR, and a large population-based study proposed a non-fasting increase of 1mmol/L in Apo-B lipoprotein remnants is associated with a 2.8-fold causal increase in risk for ischemic heart disease (60, 61). Dyslipidemia is affected by diet and lifestyle. Dietary control and improvement of living style are the basic measures to treat dyslipidemia. In addition, selective lipid-lowering medications could normalize dyslipidemia, but it hasn’t become the standard regimen for PCOS. In the future, identification of optimal subpopulation that needs lipid-lowering treatment and the efficacy of treatment in dyslipidemic PCOS women warrant further clinical research.

### Strength and Limitation

Our study exhibits the blood lipid profiles in different characteristics of PCOS in the Chinese population and discussed the possible relationships between key parameters of change and unique features of women with PCOS. We discover that dyslipidemia is associated with clinical hyperandrogenism, insulin resistance, impaired glucose metabolism, and BMI in women with PCOS. Our study also has some limitations. Firstly, due to the cross-sectional design of this study, we can’t fully systematically assess the influence of the lifestyle and type of diet on lipid profiles in PCOS women. Secondly, selection bias, such as the use of hospital-based populations, may also influence these discrepancies.

## Data Availability Statement

The raw data supporting the conclusions of this article will be made available by the authors, without undue reservation.

## Ethics Statement

Ethical approval was waived by the Ethics Committee of Obstetrics and Gynecology Hospital of Fudan University in view of the retrospective nature of the study. Written informed consent to participate in this study was provided by the participants’ legal guardian/next of kin.

## Author Contributions

FG, ZG, and LZ: collected original data; FG: analyzed the data and wrote the original draft; TF: wrote and edited the draft; XZ: supervised the study implementation. YS: conceived the idea and supervised implementation. All authors approved the final article. All authors have read and agreed to the published version of the manuscript.

## Funding

This work was supported by the Natural Science Foundation of Shanghai (No.19ZR1406700 to YS), the National Natural Science Foundation of China (No.81471438 to YS), the National Natural Science Foundation of China (No. 81871143 and 2071642 to XZ).

## Conflict of Interest

The authors declare that the research was conducted in the absence of any commercial or financial relationships that could be construed as a potential conflict of interest.

## Publisher’s Note

All claims expressed in this article are solely those of the authors and do not necessarily represent those of their affiliated organizations, or those of the publisher, the editors and the reviewers. Any product that may be evaluated in this article, or claim that may be made by its manufacturer, is not guaranteed or endorsed by the publisher.

## References

[1] Zhu T Cui J Goodarzi MO. Polycystic Ovary Syndrome and Risk of Type 2 Diabetes, Coronary Heart Disease, and Stroke. Diabetes (2021) 70(2):627–37. doi: 10.2337/db20-0800 33158931

[2] Stener-Victorin E Deng Q. Epigenetic Inheritance of Polycystic Ovary Syndrome - Challenges and Opportunities for Treatment. Nat Rev Endocrinol (2021) 17(9):521–33. doi: 10.1038/s41574-021-00517-x 34234312

[3] Mimouni N Paiva I Barbotin AL Timzoura FE Plassard D Le Gras S. Polycystic ovary syndrome is transmitted via a transgenerational epigenetic process. Cell Metab (2021) 33(3):513–30.e8. doi: 10.1016/j.cmet.2021.01.004 PMC7928942 33539777

[4] Wekker V van Dammen L Koning A Heida KY Painter RC Limpens J. Long-Term Cardiometabolic Disease Risk in Women With PCOS: A Systematic Review and Meta-Analysis. Hum Reprod Update (2020) 26(6):942–60. doi: 10.1093/humupd/dmaa029 PMC7600286 32995872

[5] Anagnostis P Tarlatzis BC Kauffman RP. Polycystic Ovarian Syndrome (PCOS): Long-Term Metabolic Consequences. Metabolism (2018) 86:33–43. doi: 10.1016/j.metabol.2017.09.016 29024702

[6] Mani H Levy MJ Davies MJ Morris DH Gray LJ Bankart J. Diabetes and Cardiovascular Events in Women With Polycystic Ovary Syndrome: A 20-Year Retrospective Cohort Study. Lancet (2013) 78(6):926–34. doi: 10.1111/cen.12068. 23046078

[7] Cibula D Cífková R Fanta M Poledne R Zivny J Skibová J. Increased Risk of non-Insulin Dependent Diabetes Mellitus, Arterial Hypertension and Coronary Artery Disease in Perimenopausal Women With a History of the Polycystic Ovary Syndrome. Hum Reprod (2000) 15(4):785–9. doi: 10.1093/humrep/15.4.785 10739820

[8] Gunning MN Fauser B. Are Women With Polycystic Ovary Syndrome at Increased Cardiovascular Disease Risk Later in Life. Climacteric (2017) 20(3):222–7. doi: 10.1080/13697137.2017.1316256 28457146

[9] Rotterdam ESHRE/ASRM-Sponsored PCOS Consensus Workshop Group Revised 2003 Consensus on Diagnostic Criteria and Long-Term Health Risks Related to Polycystic Ovary Syndrome. Fertil Steril (2004) 81(1):19–25. doi: 10.1016/j.fertnstert.2003.10.004 14711538

[10] Hatch R Rosenfield RL Kim MH Tredway D. Hirsutism: Implications, Etiology, and Management. Am J Obstet Gynecol (1981) 140(7):815–30. doi: 10.1016/0002-9378(81)90746-8 7258262

[11] Matthews DR Hosker JP Rudenski AS Naylor BA Treacher DF Turner RC. Homeostasis Model Assessment: Insulin Resistance and Beta-Cell Function From Fasting Plasma Glucose and Insulin Concentrations in Man. Diabetologia (1985) 28(7):412–9. doi: 10.1007/BF00280883 3899825

[12] Cheung LP Ma RC Lam PM Lok IH Haines CJ So WY. Cardiovascular Risks and Metabolic Syndrome in Hong Kong Chinese Women With Polycystic Ovary Syndrome. Hum Reprod (Oxford England) (2008) 23(6):1431–8. doi: 10.1093/humrep/den090 18359783

[13] Zhang J Fan P Liu H Bai H Wang Y Zhang F. Apolipoprotein A-I and B Levels, Dyslipidemia and Metabolic Syndrome in South-West Chinese Women With PCOS. Hum Reprod (2012) 27(8):2484–93. doi: 10.1093/humrep/des191 22674204

[14] Diamanti-Kandarakis E Papavassiliou AG Kandarakis SA Chrousos GP. Pathophysiology and Types of Dyslipidemia in PCOS. Trends Endocrinol Metab (2007) 18(7):280–5. doi: 10.1016/j.tem.2007.07.004 17692530

[15] Wild RA Rizzo M Clifton S Carmina E. Lipid Levels in Polycystic Ovary Syndrome: Systematic Review and Meta-Analysis. Fertil Steril (2011) 95(3):1073–9.e1-11. doi: 10.1016/j.fertnstert.2010.12.027 21247558

[16] Chen MJ Yang WS Yang JH Chen CL Ho HN Yang YS. Relationship Between Androgen Levels and Blood Pressure in Young Women With Polycystic Ovary Syndrome. Hypertension (2007) 49(6):1442–7. doi: 10.1161/HYPERTENSIONAHA.106.083972 17389259

[17] Falcetta P Benelli E Molinaro A Di Cosmo C Bagattini B Del Ghianda S. Effect of Aging on Clinical Features and Metabolic Complications of Women With Polycystic Ovary Syndrome. J Endocrinol Invest (2021) 44(12):2725–33. doi: 10.1007/s40618-021-01594-5 PMC8572193 34089497

[18] Carmina E Chu MC Longo RA Rini GB Lobo RA. Phenotypic Variation in Hyperandrogenic Women Influences the Findings of Abnormal Metabolic and Cardiovascular Risk Parameters. J Clin Endocrinol Metab (2005) 90(5):2545–9. doi: 10.1210/jc.2004-2279 15728203

[19] Chernukha C Naidukova N Udovichenko U Kaprinae KK Ivanets I. Androgen Profile in Patients With Polycystic Ovary Syndrome and its Association With Metabolic Dysfunction. Akusherstvo Ginekol (2019) 11, 122–8. doi: 10.18565/aig.2019.11.122-128

[20] Santoro N Schauer IE Kuhn K Fought AJ Babcock-Gilbert S Bradford AP. Gonadotropin Response to Insulin and Lipid Infusion Reproduces the Reprometabolic Syndrome of Obesity in Eumenorrheic Lean Women: A Randomized Crossover Trial. Fertil Steril (2021) 116:566–74. doi: 10.1016/j.fertnstert.2021.03.005 PMC8349763 33838870

[21] von Bibra H Saha S Hapfelmeier A Müller G Schwarz P. Impact of the Triglyceride/High-Density Lipoprotein Cholesterol Ratio and the Hypertriglyceremic-Waist Phenotype to Predict the Metabolic Syndrome and Insulin Resistance. Horm Metab Res (2017) 49(7):542–9. doi: 10.1055/s-0043-107782 28597452

[22] Zhang L Chen S Deng A. Association Between Lipid Ratios and Insulin Resistance in a Chinese Population. PLoS One (2015) 10(1):e0116110. doi: 10.1371/journal.pone.0116110 25635876 PMC4312024

[23] Kheirollahi A Teimouri M Karimi M Vatannejad A Moradi N Borumandnia N. Evaluation of Lipid Ratios and Triglyceride-Glucose Index as Risk Markers of Insulin Resistance in Iranian Polycystic Ovary Syndrome Women. Lipids Health Dis (2020) 19(1):235. doi: 10.1186/s12944-020-01410-8 33161896 PMC7648985

[24] Pan X Hussain MM. Gut Triglyceride Production. Biochim Biophys Acta (2012) 1821(5):727–35. doi: 10.1016/j.bbalip.2011.09.013 PMC3319358 21989069

[25] Saponaro C Gaggini M Carli F Gastaldelli A. The Subtle Balance Between Lipolysis and Lipogenesis: A Critical Point in Metabolic Homeostasis. Nutrients (2015) 7(11):9453–74. doi: 10.3390/nu7115475 PMC4663603 26580649

[26] Azziz R. Polycystic Ovary Syndrome. Obstet Gynecol (2018) 132(2):321–36. doi: 10.1097/AOG.0000000000002698 29995717

[27] Wang J Wu D Guo H Li M. Hyperandrogenemia and Insulin Resistance: The Chief Culprit of Polycystic Ovary Syndrome. Life Sci (2019) 236:116940. doi: 10.1016/j.lfs.2019.116940 31604107

[28] Hanson RW Reshef L. Glyceroneogenesis Revisited. Biochimie (2003) 85(12):1199–205. doi: 10.1016/j.biochi.2003.10.022 14739071

[29] Bjornstad P Eckel RH. Pathogenesis of Lipid Disorders in Insulin Resistance: A Brief Review. Curr Diabetes Rep (2018) 18(12):127. doi: 10.1007/s11892-018-1101-6 PMC6428207 30328521

[30] Mizuno TM. Fat Mass and Obesity Associated (FTO) Gene and Hepatic Glucose and Lipid Metabolism. Nutrients (2018) 10(11):1600. doi: 10.3390/nu10111600 PMC6266206 30388740

[31] Santini A Novellino E. Nutraceuticals in Hypercholesterolaemia: An Overview. Br J Pharmacol (2017) 174(11):1450–63. doi: 10.1111/bph.13636 PMC5429323 27685833

[32] Natali A Baldi S Bonnet F Petrie J Trifirò S Tricò D. Plasma HDL-Cholesterol and Triglycerides, But Not LDL-Cholesterol, are Associated With Insulin Secretion in non-Diabetic Subjects. Metabolism (2017) 69:33–42. doi: 10.1016/j.metabol.2017.01.001 28285650

[33] Norman RJ Masters L Milner CR Wang JX Davies MJ. Relative Risk of Conversion From Normoglycaemia to Impaired Glucose Tolerance or non-Insulin Dependent Diabetes Mellitus in Polycystic Ovarian Syndrome. Hum Reprod (2001) 16(9):1995–8. doi: 10.1093/humrep/16.9.1995 11527911

[34] Vergès B. New Insight Into the Pathophysiology of Lipid Abnormalities in Type 2 Diabetes. Diabetes Metab (2005) 31(5):429–39. doi: 10.1016/S1262-3636(07)70213-6 16357786

[35] Vergès B. Pathophysiology of Diabetic Dyslipidaemia: Where are We. Diabetologia (2015) 58(5):886–99. doi: 10.1007/s00125-015-3525-8 PMC4392164 25725623

[36] Araki E Yamashita S Arai H Yokote K Satoh J Inoguchi T. Effects of Pemafibrate, a Novel Selective PPARα Modulator, on Lipid and Glucose Metabolism in Patients With Type 2 Diabetes and Hypertriglyceridemia: A Randomized, Double-Blind, Placebo-Controlled, Phase 3 Trial. Diabetes Care (2018) 41(3):538–46. doi: 10.2337/dc17-1589 29298800

[37] Azushima K Wakui H Uneda K. Within-Visit Blood Pressure Variability and Cardiovascular Risk Factors in Hypertensive Patients With non-Dialysis Chronic Kidney Disease. Clin Exp Hypertens (2017) 39(7):665–71. doi: 10.1080/10641963.2017.1313850 28635327

[38] Lizneva D Suturina L Walker W Brakta S Gavrilova-Jordan L Azziz R. Criteria, Prevalence, and Phenotypes of Polycystic Ovary Syndrome. Fertil Steril (2016) 106(1):6–15. doi: 10.1016/j.fertnstert.2016.05.003 27233760

[39] Ollila MM Piltonen T Puukka K. Weight Gain and Dyslipidemia in Early Adulthood Associate With Polycystic Ovary Syndrome: Prospective Cohort Study. J Clin Endocrinol Metab (2016) 101(2):739–47. doi: 10.1210/jc.2015-3543 26652764

[40] Klop B Elte JW Cabezas MC. Dyslipidemia in Obesity: Mechanisms and Potential Targets. Nutrients (2013) 5(4):1218–40. doi: 10.3390/nu5041218 PMC3705344 23584084

[41] Su X Peng D. The Exchangeable Apolipoproteins in Lipid Metabolism and Obesity. Clin Chim Acta (2020) 503:128–35. doi: 10.1016/j.cca.2020.01.015 31981585

[42] Samuel VT Shulman GI. The Pathogenesis of Insulin Resistance: Integrating Signaling Pathways and Substrate Flux. J Clin Invest (2016) 126(1):12–22. doi: 10.1172/JCI77812 26727229 PMC4701542

[43] Funcke JB Scherer PE. Beyond Adiponectin and Leptin: Adipose Tissue-Derived Mediators of Inter-Organ Communication. J Lipid Res (2019) 60(10):1648–84. doi: 10.1194/jlr.R094060 PMC6795086 31209153

[44] Castoldi A Naffah de Souza C Câmara NO Moraes-Vieira PM. The Macrophage Switch in Obesity Development. Front Immunol (2015) 6:637. doi: 10.3389/fimmu.2015.00637 26779183 PMC4700258

[45] Matsuki T Horai R Sudo K Iwakura Y. IL-1 Plays an Important Role in Lipid Metabolism by Regulating Insulin Levels Under Physiological Conditions. J Exp Med (2003) 198(6):877–88. doi: 10.1084/jem.20030299 PMC2194201 12975454

[46] Kitaoka K Tsuboi A Minato-Inokawa S Honda M Takeuchi M Yano M. Determinants and Correlates of Adipose Tissue Insulin Resistance Index in Japanese Women Without Diabetes and Obesity. BMJ Open Diabetes Res Care (2020) 8(1):e001686. doi: 10.1136/bmjdrc-2020-001686 PMC7477970 32900700

[47] Odegaard JI Chawla A. Pleiotropic Actions of Insulin Resistance and Inflammation in Metabolic Homeostasis. Science (2013) 339(6116):172–7. doi: 10.1126/science.1230721 PMC3725457 23307735

[48] Zore T Joshi N Lizneva D Azziz R. Polycystic Ovarian Syndrome: Long-Term Health Consequences. Semin Reprod Med (2017) 35(03):271–81. doi: 10.1055/s-0037-1603096 28658711

[49] Sam S. Metabolic Dysfunction in Obese Hispanic Women With PCOS. Jpn J Rheumatol (2015) 10(1):58–61. 10.1093/humrep/dev073 PMC4498223 25857311

[50] Meyer C McGrath BP Teede HJ. Overweight Women With Polycystic Ovary Syndrome Have Evidence of Subclinical Cardiovascular Disease. J Clin Endocrinol Metab (2005) 90(10):5711–6. doi: 10.1210/jc.2005-0011 16046590

[51] Shroff R Kerchner A Maifeld M Van Beek EJ Jagasia D Dokras A. Young Obese Women With Polycystic Ovary Syndrome Have Evidence of Early Coronary Atherosclerosis. J Clin Endocrinol Metab (2007) 92(12):4609–14. doi: 10.1210/jc.2007-1343 17848406

[52] de Medeiros SF Yamamoto M Souto de Medeiros MA Barbosa BB Soares JM Baracat EC. Changes in Clinical and Biochemical Characteristics of Polycystic Ovary Syndrome With Advancing Age. Endocr Connect (2020) 9(2):74–89. doi: 10.1530/EC-19-0496 31905164 PMC6993261

[53] Macut D Bjekić-Macut J Savić-Radojević A. Dyslipidemia and Oxidative Stress in PCOS. Front Horm Res (2013) 40:51–63. doi: 10.1159/000341683 24002405

[54] Meun C Gunning MN Louwers YV. The Cardiovascular Risk Profile of Middle-Aged Women With Polycystic Ovary Syndrome. Clin Endocrinol (Oxf) (2020) 92(2):150–8. doi: 10.1111/cen.14117 PMC7003818 31638273

[55] Carmina E Campagna AM Lobo RA. A 20-Year Follow-Up of Young Women With Polycystic Ovary Syndrome. Obstet Gynecol (2012) 119(2 Pt 1):263–9. doi: 10.1097/AOG.0b013e31823f7135 22270277

[56] Elting MW Korsen TJ Rekers-Mombarg LT Schoemaker J. Women With Polycystic Ovary Syndrome Gain Regular Menstrual Cycles When Ageing. Hum Reprod (2000) 15(1):24–8. doi: 10.1093/humrep/15.1.24 10611183

[57] Brown ZA Louwers YV Fong SL. The Phenotype of Polycystic Ovary Syndrome Ameliorates With Aging. Fertil Steril (2011) 96(5):1259–65. doi: 10.1016/j.fertnstert.2011.09.002 21963227

[58] Wild RA Carmina E Diamanti-Kandarakis E. Assessment of Cardiovascular Risk and Prevention of Cardiovascular Disease in Women With the Polycystic Ovary Syndrome: A Consensus Statement by the Androgen Excess and Polycystic Ovary Syndrome (AE-PCOS) Society. J Clin Endocrinol Metab (2010) 95(5):2038–49. doi: 10.1210/jc.2009-2724 20375205

[59] Crumbach ME Krgati L Golua T Baldani DP. Long-Term Consequences of Polycystic Ovary Syndrome. Gynaecol Perinatol (2016) 25(2):53–8.

[60] Vine DF Wang Y Jetha MM Ball GD Proctor SD. Impaired ApoB-Lipoprotein and Triglyceride Metabolism in Obese Adolescents With Polycystic Ovary Syndrome. J Clin Endocrinol Metab (2017) 102(3):970–82. doi: 10.1210/jc.2016-2854 27997268

[61] Varbo A Benn M Tybjærg-Hansen A Jørgensen AB Frikke-Schmidt R Nordestgaard BG. Remnant Cholesterol as a Causal Risk Factor for Ischemic Heart Disease. J Am Coll Cardiol (2013) 61(4):427–36. doi: 10.1016/j.jacc.2012.08.1026 23265341

